# Müller glia–mediated regeneration restores neuronal diversity and retinal circuit organization in the adult zebrafish

**DOI:** 10.64898/2026.03.15.711785

**Published:** 2026-03-17

**Authors:** Mikiko Nagashima, Lara Rappaport Da Costa Santos, Sherine Awad, Zachary Flickinger, Peter F. Hitchcock, Thanh Hoang

**Affiliations:** 1 Department of Ophthalmology and Visual Sciences, University of Michigan School of Medicine, Ann Arbor, MI 48105, USA; 2 Department of Cell and Developmental Biology, University of Michigan School of Medicine, Ann Arbor, MI 48105, USA.; 3 Michigan Neuroscience Institute, University of Michigan School of Medicine, Ann Arbor, MI 48105, USA.

## Abstract

The ability to regenerate neurons with the appropriate identities and connectivity is a major challenge in regenerative neuroscience. Unlike mammals, zebrafish can regenerate retinal neurons after injury through reprogramming of endogenous Müller glia. However, it remains unclear how closely regenerated neurons match the identities, diversity and structural features of the cells that were lost. Here, we combined inducible lineage tracing, single-cell RNA sequencing and morphological analysis to define the molecular and structural features of Müller glia–derived neurons in the adult zebrafish retina. Using light lesion and NMDA injury to selectively ablate photoreceptors or inner retinal neurons, we found that injury context biased the relative proportions of regenerated neurons, with each paradigm favoring replacement of the populations most affected by damage. Nevertheless, both injury paradigms generated all major retinal cell classes, indicating that Müller glia–derived progenitors retain broad neurogenic potential. Regenerated neurons showed substantial transcriptional similarity to endogenous counterparts, with the remaining differences primarily reflecting ongoing maturation. At subtype resolution, regenerated amacrine cells restored broad neurochemical and morphological diversity, and regenerated retinal ganglion cells re-established long-range projections to the optic tectum. Together, these findings show that Müller glia–mediated regeneration in the adult zebrafish retina restores neuronal diversity and key features of retinal circuit organization, providing insights for understanding how complex neuronal identities and connectivity can be rebuilt after injury.

## Introduction

The development of the nervous system, such as the vertebrate retina, relies on the orchestrated differentiation of multipotent progenitors into diverse types of neurons and glia, and the integration of neurons into precise synaptic circuits (Agathocleous and Harris 2009; Cepko 2014; Götz and Huttner 2005). This developmental program is governed by temporal and spatial cues that regulate progenitors’ transition from proliferation to cell-cycle exit, cell fate determination, and circuit assembly (Götz and Huttner 2005; Livesey and Cepko 2001). In the adult, neuronal regeneration faces significant challenges, as neurogenic programs must be reactivated within mature tissue where architecture and connectivity are already established, and newly generated neurons need to adopt appropriate subtype identities and integrate into existing circuits.

The mammalian central nervous system possesses a limited capacity to regenerate neurons (Ramón y Cajal 1991; Richardson et al. 1980). Neuronal loss due to injury or disease typically results in permanent functional deficits. Nonetheless, recent advancements in the field of regeneration biology have revised this long-standing view by demonstrating that endogenous glia possess latent neurogenic potential and can be induced to generate new neurons in the adult brain. For instance, subsets of astrocytes have been successfully reprogrammed into neurons in the brain through forced expression of proneuronal transcriptional factors (Götz et al. 2015; Yin et al. 2025). Similarly, in the mouse retina, Müller glia, which typically remain quiescent or undergo reactive gliosis following injury, can be induced to generate neurons (Jorstad et al. 2017; Todd et al. 2022; Yang et al. 2025; Hoang et al. 2020; Le et al. 2024). However, two central barriers remain for potential therapeutic translation: the limited diversity of regenerated neurons and the uncertain extent to which regenerated neurons acquire the mature transcriptional programs, morphologies, and circuit-level connectivity required for functional restoration. Addressing these gaps requires comparisons of regenerated neurons to their endogenous counterparts at both molecular and structural levels.

Unlike mammals, the teleost fish retina possesses a remarkable capacity to regenerate retinal neurons via reprogramming of Müller glia into stem cell state (Lenkowski and Raymond 2014; Karl and Reh 2010; Hamon et al. 2016);(Bernardos et al. 2007). Following injury or cell death, Müller glia de-differentiate, enter the cell cycle, and divide asymmetrically to produce multipotent Müller glia-derived progenitors (Nagashima et al. 2013; Lenkowski and Raymond 2014). These progenitors proliferate and can give rise to each of the major retinal cell types (Powell et al. 2016; Lyu et al. 2023; Lahne, Brecker, et al. 2020). While the molecular mechanisms governing the initial reprogramming response of Müller glia have been increasingly well-characterized (Lahne, Nagashima, et al. 2020; Hoang et al. 2020; Nagashima and Hitchcock 2021; Jui and Goldman 2024), the precise identity of regenerated neurons remains poorly characterized. Specifically, it remains unclear whether Müller glia–derived progenitors are instructed to replace the specific neuronal types lost to injury or instead follow a more intrinsic default neurogenic program. Furthermore, the degree to which regenerated neurons recapitulate the distinct transcriptional signatures, complex morphologies, and cellular heterogeneity equivalent to endogenous counterparts, remains unknown. Given that the retina is characterized by an extraordinary degree of subtype heterogeneity, the functional restoration of vision requires the precise reconstruction of the diverse neuronal landscape. Verifying these characteristics is critical to determine whether regenerative neurogenesis can successfully reconstitute a functional retina.

Here, we utilized an inducible genetic lineage-tracing strategy to definitively label Müller glia-derived regenerated neurons. This allows us to distinguish and isolate regenerated neurons from surviving populations. By combining this lineage-tracing approach with single-cell transcriptomics (scRNA-Seq) and high-resolution morphological analyses, we performed comprehensive characterization of Müller glia-derived neurons. Using two injury paradigms that preferentially ablate different neuronal populations: light lesion induced photoreceptor loss and NMDA-induced excitotoxicity targeting inner retinal neurons, we directly compare regenerative outcomes across damage contexts. Our analyses show that injury type biases the relative proportions of regenerated neurons, yet both paradigms produce a broad range of retinal cell types. This suggests that Müller glia-derived progenitors follow a robust, intrinsic neurogenic program, which may be conserved from developmental programs. Transcriptomic analysis shows that regenerated neurons largely overlap with endogenous counterparts, indicating successful restoration of homeostatic molecular signature. Finally, using subtype analysis of amacrine cells, we show that zebrafish retinas regenerate diverse amacrine neuron subtypes with distinct transcriptional, neurochemical, and morphological profiles. Together, these findings indicate that adult zebrafish retinal regeneration can restore complex neuronal identities at molecular and structural levels, providing insight into how regenerative neurogenesis achieves precise circuit reconstruction in the mature nervous system.

## Results

### 1. Genetic lineage tracing of Müller glia-derived neurons following retinal injuries using the mmp9:CreERT2 line

To assess the identity of regenerated retinal neurons after selective cell ablation, we first validated the cell type specificity of our injury paradigms. Since previous work shows that prolonged light exposure damages inner retinal neurons in addition to photoreceptors (Lyu et al. 2023), we performed TUNEL apoptotic assays to determine whether our acute high-intensity light lesion model avoided collateral damage. TUNEL assays performed immediately following light exposure revealed a robust accumulation of apoptotic cells confined in the outer nuclear layers (ONL) that peaked between 1 and 2 days post lesion (Fig. S1A,B). In contrast, no TUNEL+ cells were observed in the inner nuclear layer (INL) or ganglion cell layer (GCL) (Fig. S1A,B), and the number of HuC/D+ neurons remained unchanged (Fig. S1C,D). Conversely, intravitreal NMDA injection selectively ablated inner retinal neurons without detectable damage to the ONL (Fig. S1E,F). These data confirm that our two injury models selectively ablate distinct retinal neuronal types.

**Figure 1.**
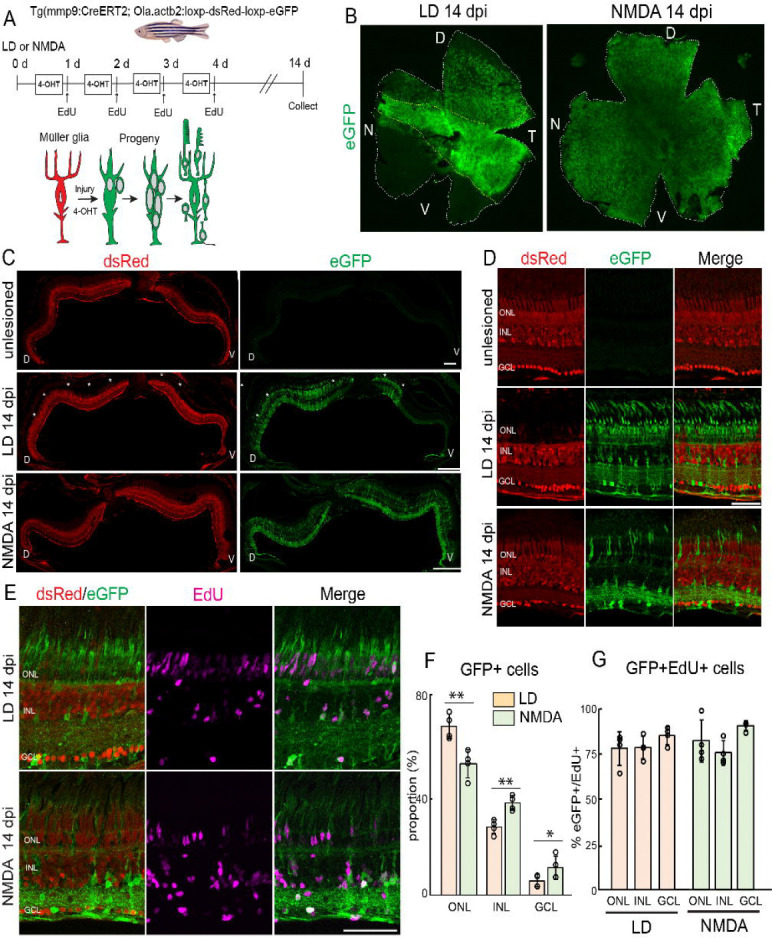
Injury-inducible lineage tracing specifically labels Müller glia-derived progeny. (A) Breeding strategy and experimental paradigm of retinal lesions, 4-hydroxitamoxifen treatment and EdU injection to label Müller glia-derived progeny using Tg(mmp9:creERT2; Ola.actb:lox-p-dsRed-loxp-eGFP) transgenic lineage tracing line. (B) Flat-mount whole retinas immunostained for eGFP at 14 days post light lesion and NMDA injection. (C, D) Retinal cross sections immunostained for eGFP in unlesioned, 14 days post light and NMDA damage. Asterisks indicate damaged photoreceptor regions. (E) Retinal cross sections immunostained for eGFP and EdU at 14 days post light (top) and NMDA (bottom) damage. (F) Quantification of the proportion of the eGFP+ neurons in light-lesioned and NMDA-injected retinas in the different retinal layers. (G) Quantification of the percentage of eGFP/EdU double positive cells from total EdU+ cells in light-lesioned and NMDA-injected retina within the different retinal layers. Scale bars: C 200 um; D, G 50 um; 4-OHT: 4-hydroxitamoxifen; ONL:outer nuclear layer; INL: inner nuclear layer; GCL ganglion cell layer; LD: light lesion; NMDA: N-methyl-D-aspartate; dpl: days post lesion; dpi: days post injection; D: dorsal; V: ventral; N: nasal; T: temporal; EdU: 5-Ethynyl-2)-deoxyuridine.

To unambiguously distinguish regenerated neurons from endogenous cells, we employed inducible Cre-loxP–based genetic lineage tracing. We generated a transgenic zebrafish line by crossing *Tg(mmp9:CreERT2) with Tg(Ola.actb2:loxp-dsRed-loxp-eGFP)* (Fig. 1A) (Ando et al. 2017). In this system, the Medaka actin2 promoter drives ubiquitous dsRed expression in all retinal cells under normal conditions. Following retinal injury, mmp9 expression is specifically induced in reprogrammed Müller glia and their progenitors (Silva et al. 2020; Lyu et al. 2023). Administration of 4-hydroxytamoxifen (4-OHT) after retinal lesion therefore induces CreERT2-mediated recombination selectively in activated Müller glia, permanently switching reporter expression from dsRed to eGFP in lineage-traced cells (Fig. 1A). As expected, unlesioned retinas showed strong dsRed signal without detectable eGFP (Fig. 1C,D).

To establish the specificity and inducibility of the *mmp9:CreERT2* system, we performed two control experiments. Treatment with 4-OHT in the absence of injury did not induce eGFP expression (Fig. S2A), confirming that the mmp9 promoter is inactive in uninjured retinas, and that CreERT2 does not exhibit leaky activation. Conversely, light lesions without 4-OHT administration did not induce eGFP despite robust endogenous mmp9 upregulation (Fig. S2B), demonstrating that CreERT2-mediated recombination strictly requires 4-OHT ligand activation. Together, these control experiments demonstrate that the *Tg(mmp9:CreERT2; Ola.act2; loxp-dsRed-loxp-eGFP)* system shows no detectable background recombination and provides tightly regulated, injury-dependent lineage labeling.

We next characterized the temporal dynamics of Müller glial lineage labeling following light lesion. This paradigm was selected due to its well-defined and highly synchronized response of Müller glia (Vihtelic and Hyde 2000). At 3 days post lesion (dpi), eGFP+ cells appeared as radial columns spanning the retinal thickness (Fig. S2C). These eGFP+ cells co-expressed the Müller glial marker glutamine synthetase (GS), confirming selective labeling of injury-activated Müller glia (Fig. S2C). Müller glia–derived progenitors appeared as elongated nuclei associated with radial processes. The intensity of eGFP signal in Müller glia-derived progenitors was relatively weaker, likely due to delayed reporter accumulation. Between 3 to 7 dpI, Müller glia-derived progenitors expand and migrate into the different retinal layers (Bernardos et al. 2007; Nagashima et al. 2013). By 7 dpi, subsets of eGFP+ cells in the ONL expressed cone (Zpr1) and rod (Zpr3) photoreceptor markers (Fig. S2D,E), indicating progression toward photoreceptor differentiation. These regenerated photoreceptors displayed immature morphology, including shortened outer segments, consistent with ongoing maturation. Together, these data demonstrate that the mmp9-driven lineage tracing system faithfully captures the progression from Müller glial activation through progenitor expansion to neuronal differentiation.

We next examined regenerated neurons at 14 dpi and compared regenerative outcomes between light lesion and NMDA injury. Retinal flat-mount analysis revealed spatial correlation between damage pattern and eGFP+ regenerated neurons. Acute light lesion induces photoreceptor death within a central horizontal band along the nasal-temporal axis, with partial dorsal spread and sparing of the ventral retina (Qin et al. 2009; Nagashima et al. 2013). At 14 dpi, the distribution of eGFP+ regenerated neurons mirrored this pattern, with strong signal in the central band, sparse labeling dorsally, and no detectable signal ventrally (Fig.1B,C). In contrast, NMDA injury resulted in a broader distribution of eGFP+ cells across the retina (Fig. 1B,C), consistent with widespread loss of the inner neurons. Consistent with these patterns, light-lesioned retinas showed enrichment of eGFP+ cells in the ONL, along with additional labeled neurons in the INL and GCL (Fig. 1C,D,F). In contrast, NMDA-injected retinas exhibited more prominent eGFP labeling in the INL and GCL (Fig. 1C,D,F). These findings confirm that the lineage tracing system faithfully labels regenerated neurons in patterns consistent with injury location.

Finally, to verify that eGFP+ neurons originate from proliferating Müller glia, we performed EdU incorporation assays during the early regenerative window (1–4 dpi; Fig. 1E). At 14 dpi, 75.6% (light lesion) and 77.7% (NMDA) of eGFP+ cells were EdU+ (Fig. 1G), with comparable proportions across retinal layers. EdU+/eGFP− cells likely represent activated microglia or rod photoreceptors derived from eGFP− rod precursors (Stenkamp 2011; Nagashima et al. 2013; Mitchell et al. 2018). The high fraction of EdU+/eGFP+ cells confirms that the majority of lineage-traced neurons arise from proliferative Müller glia–derived progenitors.

### 2. Injury context biases the proportion of regenerated neurons but does not restrict neuronal diversity

**Figure 2.**
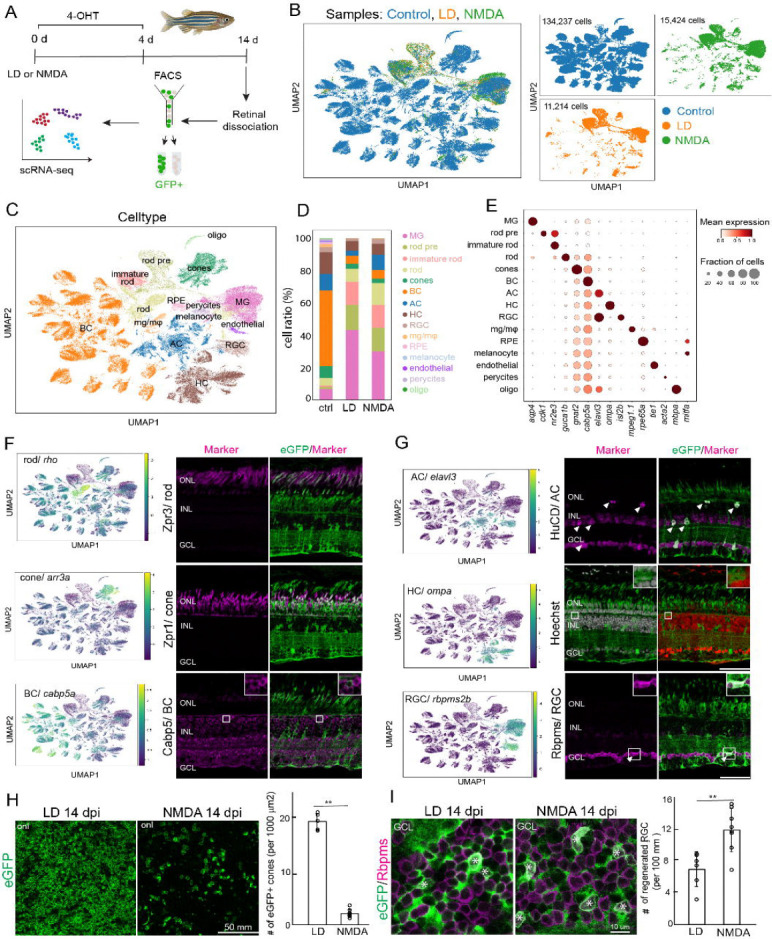
Regeneration restores all major retinal classes across injury paradigms. (A). Experimental design and scRNA-Seq profiling of FACS-isolated eGFP+ cells after injuries. (B) UMAP plots showing the single cell distribution of control, light-lesioned, and NMDA-injected samples. (C) Cell type annotation on UMAP plot across all samples. (D) Quantification of the ratios of retinal cells in control, light-lesioned and NMDA-injected samples. (E) Dot plot of selected markers of major retinal cell types. (F) scRNA-Seq mRNA expression and representative immunohistochemistry for rod photoreceptors (rho), cone photoreceptor markers (arr3a and Zpr1) and bipolar cells (calbp5a) at 14 days post light-lesioned retinas. (G) scRNA-Seq mRNA expression and representative immunohistochemistry for amacrine cells (elavl3), horizontal cells (ompa) and RGCs (rbpms2a) at 14 days post light lesion. (H) Representative immunostained images and quantification of eGFP-labeled cones in the outer retinal layers from flat-mount retinas at 14 days post light lesion or NMDA-injection. (I) Representative immunostained images and quantification of eGFP and Rbpms at retinal ganglion cell layer from flat-mount retinas at 14 days post light or NMDA damage. LD: light lesion; NMDA: N-methyl-D-aspartate; dpl: days post lesion; BC: bipolar cells; AC: amacrine cells; HC: horizontal cells; ONL:outer nuclear layer; INL: inner nuclear layer: GCL: ganglion cell layer. Scale bars 50 um.

To determine how precisely regeneration matches the identities of ablated neurons, we performed single-cell RNA sequencing (scRNA-Seq) on FACS-isolated eGFP+ cells derived from Müller glia at 14 dpi following either light lesion or NMDA injection (Fig.2A). After cell filtering and quality control, we retained 11,214 cells from light-lesioned retinas and 15,424 cells from NMDA-injected retinas (Fig.2B). For unlesioned control, we incorporated our previous scRNA-Seq datasets comprising 134,237 cells (Lyu et al. 2025). Unsupervised clustering and annotation using established marker genes identified all major retinal cell classes in both lesion paradigms (Fig. 2C–E), including photoreceptors, bipolar cells, amacrine cells, horizontal cells, and retinal ganglion cells (RGCs).

We next validated these scRNA data findings by immunohistochemical analysis. In light-lesioned retinas, abundant eGFP-labeled photoreceptors were detected in the ONL and co-labeled with rod (Zpr3) or cone (Zpr1) markers (Fig. 2F). As expected, NMDA-injured retinas contained fewer regenerated rod and cone photoreceptors (Fig. S3). These regenerated photoreceptors display characteristic morphology, including the formation of outer segments (Fig. 2F, Fig. S3). In both injury paradigms, eGFP+ bipolar cells (Cabp5+), amacrine cells (HuC/D+), horizontal cells (identified by position and morphology) and RGCs (Rbpms) were detected (Fig. 2F,G; Fig. S3), confirming the regeneration of inner retinal neurons. Thus, regardless of injury type, Müller glia–derived progenitors generated a broad spectrum of retinal neuronal types.

We next compared the relative proportion of regenerated neurons between the two injury models. As expected, the scRNA data showed that NMDA injury increased the regeneration of inner retinal neurons, including amacrine cells (LD 3.05% vs NMDA 9.23%) and RGCs (LD 1.4% vs NMDA 2.96%) (Fig. 2D), consistent with the immunohistochemistry data (Fig. 1D,F). However, the ratio of cone photoreceptors is comparable between both paradigms (LD 3.32%; NMDA 2.75%), and NMDA samples contained a high frequency of rod photoreceptors (LD 7.9%; NMDA 13.74%). To quantify cone regeneration more precisely, we analyzed regenerated cones in flat-mount retinal preparations. Our analysis prioritized the cone population because cones arise directly from Müller glia-derived progenitors during the primary regenerative phase (Lenkowski and Raymond 2014), and their well-characterized profiles allow for highly accurate cell count (Nagashima et al. 2017, 2020). In contrast, rod photoreceptors may be continuously generated from eGFP-labeled rod precursors (Stenkamp 2011), which can confound the analysis of the acute regenerative response. Light-lesioned retinas exhibited significantly greater cone regeneration compared to NMDA-injected retinas (Fig. 2H). In contrast, NMDA injection resulted in a two-fold increase in the number of regenerated RGCs than in light lesion (Fig. 2I). Collectively, these data demonstrate injury context biases the fate of Müller glia-derived progenitors toward neuronal types originally ablated, yet does not restrict overall neuronal diversity. This implies that Müller glia-derived progenitors are responsive to environmental cues, however, injury extrinsic signals do not override a robust intrinsic program of generating all retinal neuron types.

### 3. Regenerated neurons transcriptionally resemble endogenous counterparts

**Figure 3.**
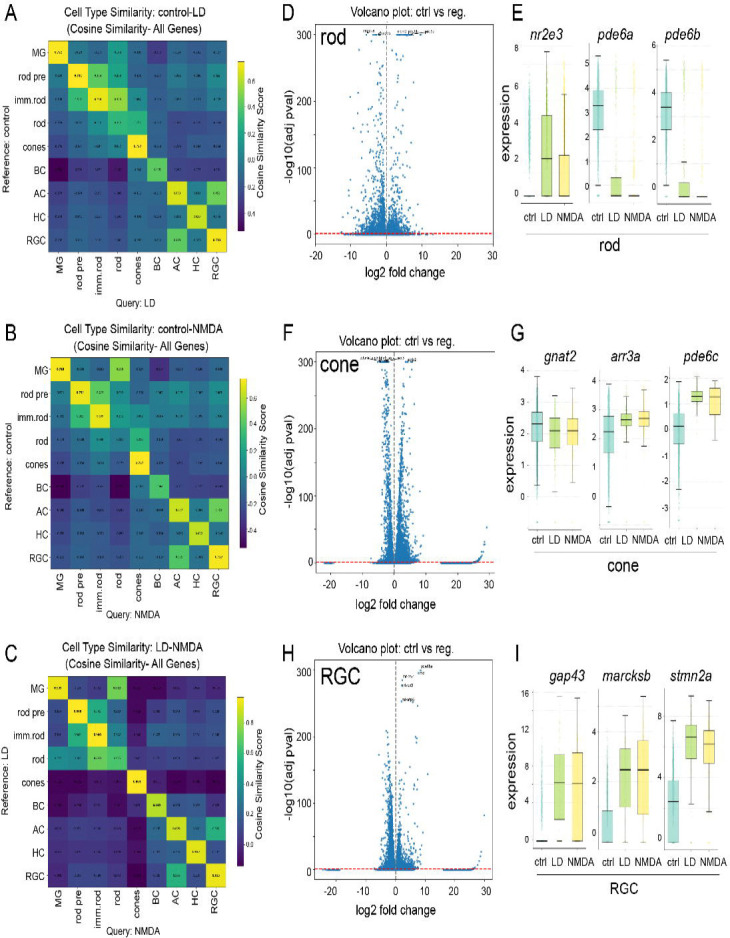
Regenerated neurons closely match endogenous transcriptional identities (A-C) Cosine similarity analysis between control and light lesion (A), control and NMDA injection (B), and light lesion and NMDA injection (C). (D-I) Volcano plots (D, F, H) and box plots for selected genes (E, G, I) of cone (D, E) and rod (F,G) photoreceptors and retinal ganglion cells (H,I).

We next assessed to what extent the regenerated neurons transcriptionally resemble endogenous retinal neurons. scRNA-Seq UMAP visualization revealed substantial overlap between regenerated and unlesioned populations across all major retinal classes (Fig. 2B,C), suggesting that regenerated neurons have acquired transcriptional states similar to their homeostatic counterparts. We next performed the transcriptional similarity analysis (Watson et al. 2022) to quantitatively analyze the overall consistency of the transcriptome in all sample conditions. In both injury paradigms, regenerated cell types show high degrees of transcriptional similarity with native cells, including Müller glia, rod precursors/immature rod, cones, amacrine, horizontal cells and RGCs (Fig. 3A–C). Meanwhile, regenerated rods are more similar to immature rods. The similarity was highest between light-lesioned and NMDA-injected samples (Fig. 3A–C), consistent with activation of a shared intrinsic differentiation program independent of lesion models.

Differential gene expression analysis revealed relatively few differences between regenerated and endogenous neurons. Inner retinal neurons, including bipolar, amacrine, and horizontal cells, exhibit largely similar core transcriptional programs and neurotransmitter identity genes between regenerated neurons and endogenous counterparts (Fig. S4). Likewise, regenerated cone photoreceptors similarly exhibited mature phototransduction gene expression comparable to unlesioned controls (Fig. 3D,E). In contrast, regenerated rod photoreceptors displayed immature states, including elevated expression of the immature rod marker *nr2e3* and reduced levels of mature phototransduction genes (Fig. 3F,G, Fig.S4A). This is consistent with the known ontogeny of rods in the teleost retina, where rod precursors continue to proliferate and differentiate throughout life. Indeed, proliferative rod precursors were detected within regenerated samples (Fig. 2C,D), suggesting ongoing rod neurogenesis at 14 dpi underlies the lower transcriptomic similarity between unlesioned and regenerated rods.

Similarly, regenerated RGCs upregulated genes associated with axonal growth, cytoskeletal remodeling, and guidance (Fig. 3H,I), while maintaining stable expression of core identity transcription factors and neurotransmitter synthesis genes (Fig. S4H). These transcriptional signatures suggest that regenerated RGCs are specified but remain in maturation, extending their axons and reestablishing their connectivity. Collectively, integrated transcriptomic and histological analyses demonstrate that injury context biases relative proportions, yet neuronal diversity is broadly preserved, and regenerated neurons largely recapitulate endogenous molecular identities, with minor differences primarily reflecting ongoing maturation.

### 4. Regenerated amacrine cells reconstitute subtype diversity and neurochemical identity.

**Figure 4.**
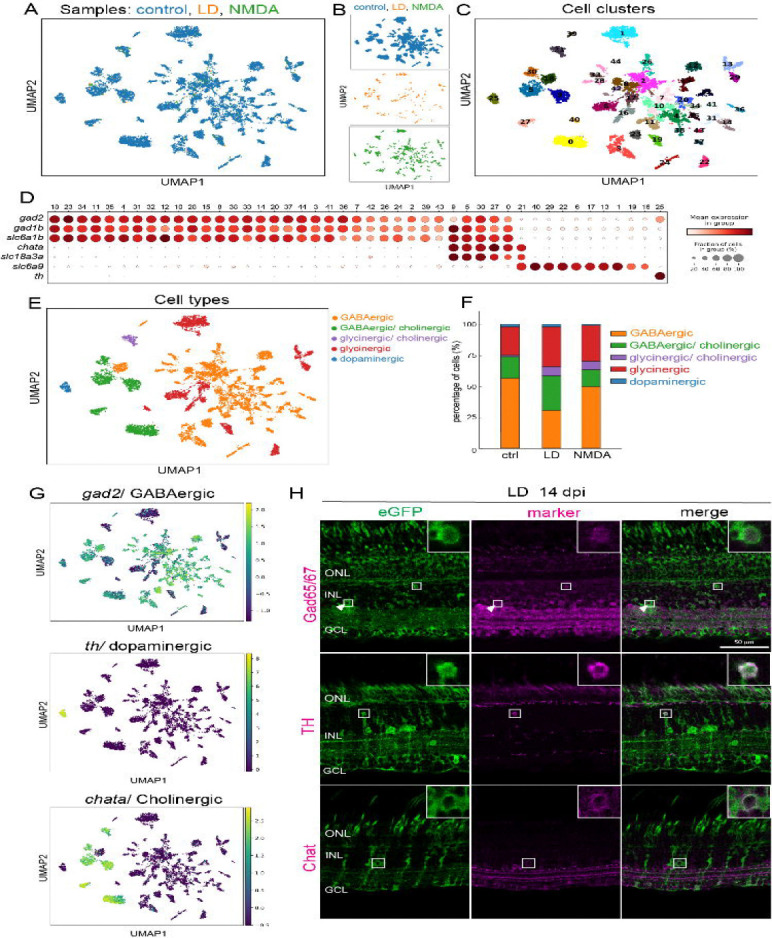
Regeneration restores subtype diversity among amacrine cells (A, B) UMAP plots showing amacrine cell populations from control, light-lesioned and NMDA-injected sample groups. (C) UMAP plot showing regenerated amacrine cell clusters across the three sample groups. (D) Dot plot showing the expression of selected markers for neurotransmitter-defined amacrine cell subtypes (GABAergic: gad2, gad1b, slc6a1b; Cholinergic: chata, slc18a3a; glycinergic: slc6a9; dopaminergic: th. (E) Annotation of main subtypes of amacrine cells. (F) Proportion of main amacrine subtypes across the three sample groups. (G, H) Feature plots and representative immunostained images of selected amacrine subtype markers, Gad65/67, TH, and Chat at day 14 after light lesions. Scale bars: 50 um. LD: light lesion; onl:outer nuclear layer; inl: inner nuclear layer: GCL ganglion cell layer.

In the vertebrate retina, each major cell type encompasses transcriptionally and neurochemically distinct subtypes with specialized physiological roles (Masland 2012a).This cellular diversity is fundamental for visual function and acuity (Masland 2012a; Wässle 2004). Restoring retinal function requires not only regeneration of major cell classes but also subtype-level diversity and circuit-specific features. Amacrine cells represent one of the most heterogeneous retinal populations, defined by distinct transcriptional programs, neurotransmitter identities and laminar-specific dendritic architecture within the IPL (Connaughton et al. 2004; Masland 2012b; Jusuf and Harris 2009). While at least 63 transcriptionally distinct amacrine subtypes have been identified in mice (Yan et al. 2020), it remains unknown in zebrafish retina. We therefore focused on amacrine cells to assess the cell diversity and precision of regenerative neurogenesis. Sub-clustering analysis of the amacrine cell population (control: 10,035 cells; light lesion: 236 cells; NMDA: 867 cells) identified 45 transcriptionally distinct clusters (Fig. 4A–C; Fig. S5). Each cluster is defined by unique molecular signatures, representing both known and potentially novel amacrine subtypes. NMDA-injured retinas contained all 45 clusters, whereas light-lesioned retinas exhibited 32 clusters, likely reflecting lower sampling depth. These data indicate that Müller glia–derived progenitors regenerate a broad spectrum of amacrine subtypes across injury paradigms.

We next classified amacrine clusters based on canonical neurotransmitter markers: GABAergic (gad2, gad1b, slc6a1b), glycinergic (slc6a9), cholinergic (chata, slc18a3), and dopaminergic (th). Consistent with previous studies, all identified subtype clusters express markers for the inhibitory transmitters, either GABA or glycine, in mutually exclusive patterns (Fig. 4D, Fig.S5A,B). Of the 45 clusters, 35 were GABAergic. and 10 were glycinergic. Cholinergic clusters co-expressed either GABAergic (five clusters) or glycinergic (one cluster) markers. Unlike mouse retinas, which possess at least two dopaminergic amacrine subtypes(Yan et al. 2020), only one *th*-expressing cluster was identified in zebrafish retina (Fig. 4D). Notably, all major neurochemical classes were regenerated in both lesion paradigms (Fig. 4E,F; Fig. S5A–C).Immunohistochemistry confirmed the presence of regenerated GABAergic (Gad65/67+), cholinergic (Chat+), and dopaminergic (TH+) amacrine cells in both light-lesioned (Fig.4G,H) and NMDA-injected retinas (Fig.S6), validating the transcriptomic classification.

Although subtype diversity was broadly restored, relative proportions differed modestly from unlesioned retinas. Regenerated samples exhibited a relative increase in glycinergic (control: 24%, LD: 40%; NMDA: 36%) and cholinergic (control 19%; LD 36%; NMDA 21%) populations and a corresponding decrease in GABAergic cells (control: 74%, LD: 58%; NMDA: 63%), while dopaminergic proportions remained similar (Fig. 4E,F; Fig. S6). Given the limited number of regenerated amacrine cells recovered for scRNA-Seq, these proportional shifts should be interpreted cautiously. Nonetheless, these results indicate that regenerated amacrine cells acquire not only appropriate transcriptomic features but also appropriate neurochemical identities, suggesting that the regenerative capacity of the adult zebrafish extends to the production of complex, diverse interneuron subtypes.

### 5. Regenerated amacrine cells re-acquire stereotyped dendritic morphology and circuit integration

Amacrine subtype identity is defined not only by transcriptional and neurochemical features but also by highly stereotyped dendritic morphology and IPL stratification, which determine synaptic connectivity (Connaughton et al. 2004; Masland 2012b; Jusuf and Harris 2009). Since cell morphology dictates synaptic connectivity and function, structural recovery of regenerated cells can be utilized as a proxy for the re-establishment of the functional integration of regenerated neurons into synaptic circuits. We therefore examined whether regenerated amacrine subtypes re-establish characteristic dendritic architectures. We focused on examining the dendritic arborization and stratification pattern of two morphologically distinct and well-defined subtypes: cholinergic amacrine cells and dopaminergic amacrine cells, in the dorsal regions of light-lesioned retinas, where eGFP+ cells are sparse (Fig. 1B,C).

**Figure 5.**
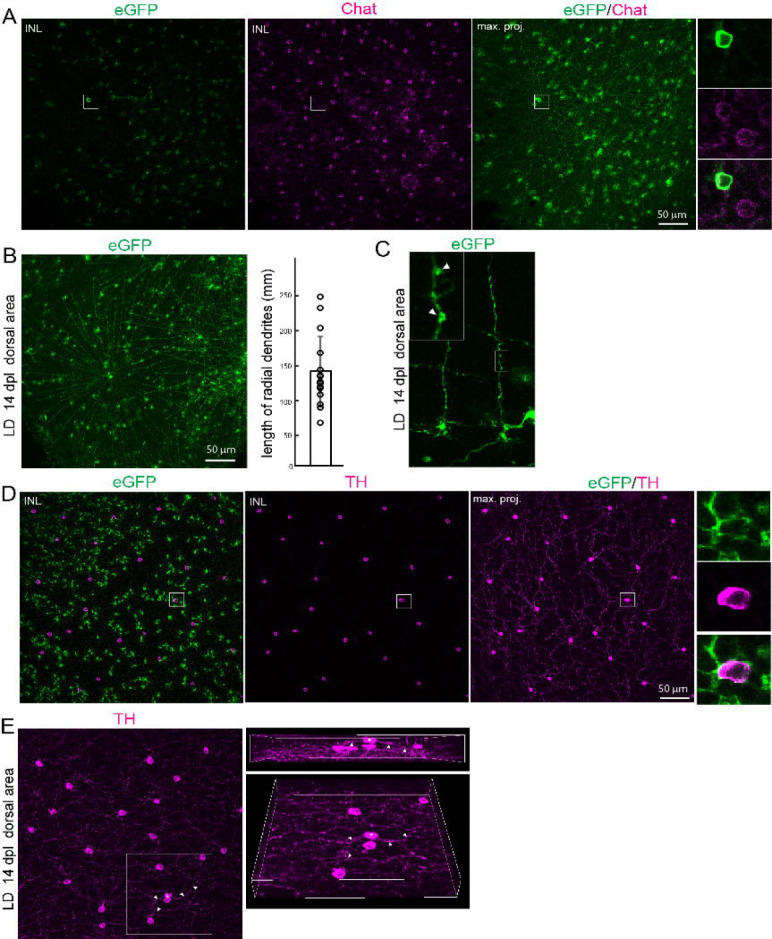
Regenerated amacrine subtypes regain characteristic morphology and laminar organization. (A) Flat-mount retinas at day 14 post light lesions immunostained for eGFP and Chat. A single optical section (left and middle) at the inner nuclear layer and Z-stack maximum projection showing Chat+ starburst amacrine cells. (B) eGFP+ regenerated starburst amacrine cells and quantification of the length of radial dendrites in regenerated starburst amacrine cells. (C) High magnification image of starburst amacrine processes on retinal cross sections. The boxed region represents varicosity. (D) Flat-mount retinas at day 14 post light lesions immunostained for eGFP and TH. A single optical section (left and middle) at the inner nuclear layer and Z-stack maximum projection showing cell bodies of TH+ cells and their dendritic axons within the inner plexiform layer. (E) Flat-mount preparation of light-lesioned retina immunostained for TH. Z-stack maximum projection of TH-labeled cells and their dendritic axons. Asterisk indicates apically displaced TH-labeled cells extending their dendritic processes (arrows) extending toward the inner plexiform layer. Scale bars: 50 um. INL: inner nuclear layer.

Cholinergic starburst amacrine cells are characterized by a radially symmetric dendritic tree radiating outward from the central cell body and stratified within the specific sublamina of the IPL (Famiglietti 1983). Their primary function is to provide the inhibitory foundation of direction selectivity, allowing the visual system to distinguish the direction of moving objects (Fried et al. 2002; Vaney et al. 2012). Because their stereotyped structure is the primary driver of their specialized visual function, examining the morphology of regenerated starburst amacrine cells is critical to test their potential to restore motion detection. In the flat-mount preparation of the lineage tracing line, we found abundant eGFP+ cells exhibiting the classical “starburst” morphology, with dendrites radiating from the central soma (Fig. 5A,B). Co-labeling with cholinergic marker, Chat, confirmed their cholinergic identity (Fig. 5A,B). Their dendritic field diameters range from 63.3um to 245.2um (Ave 136.7±104.1; Fig. 5B). The distal region of dendrite contains numerous varicosities (Fig. 5C, arrows), suggesting the formation of functional synapses. These findings indicate that regenerated starburst cells acquire hallmark morphological features associated with functional direction selectivity.

In the retina, dopaminergic amacrine cells are among the most sparsely distributed but functionally important amacrine subtypes (Keeley and Reese 2010). Our scRNA-Seq analysis shows that dopaminergic amacrine cells represent about 1% of total amacrine population and constitute a single subtype (Fig. 4E, F and Fig. S5). Their cell bodies reside in the inner portion of the INL and extend long dendrites (Keeley and Reese 2010). In teleost fish, not mammals, dopaminergic amacrine cells are known as interplexiform cells, extending their process toward the inner plexiform layer and the outer plexiform layer (Dowling and Ehinger 1978; Li and Dowling 2000). Our analysis prioritized processes extending toward the inner plexiform layer, as their dendritic meshwork can be unambiguously traced back to the parent somata. eGFP lineage tracing combined with TH immunostaining identified regenerated dopaminergic neurons in flat-mount retinas (Fig. 5D). These cells extended fine dendritic processes toward the IPL and exhibited clear sublaminar organization. Although ectopic regenerated dopaminergic amacrine cells were occasionally observed within the outer region of the INL, their processes also projected toward and integrated within the existing IPL meshwork (Fig. 5E). Little to no evidence of aberrant connectivity of regenerated dopaminergic neurons suggests a high degree of successful structural integration. Taken together, these data demonstrate that Müller glia–derived progenitors regenerate not only molecularly diverse amacrine subtypes but also re-establish their dendritic architecture and laminar organization. Thus, adult zebrafish retinal regeneration restores interneuron diversity at transcriptional, neurochemical and structural levels, closely recapitulating developmental programs.

### 6. Regenerated RGCs show incomplete laminar precision but re-establish long-range axonal projections

**Figure 6.**
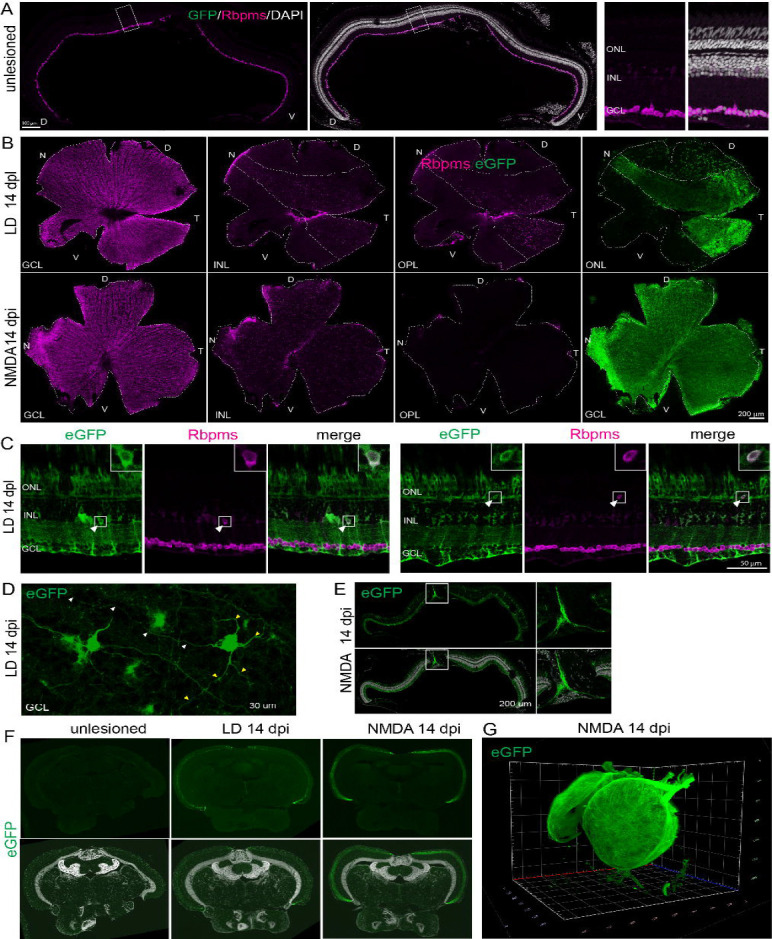
Regenerated RGCs show incomplete laminar precision but re-establish long-range projections. (A) Immunocytochemistry for RGC marker, Rbpms, on unlesioned retinal sections. (B) Serial images at different retinal layers of flat-mounted retinas immunostained for eGFP and Rbpms at 14 days post light and NMDA damages. (C) Representative images immunostained for eGFP and Rbpms in light-lesioned retinas, showing examples of mislocated regenerated RGCs. (D) Flat-mount retinal images showing eGFP-labeled regenerated RGCs extend their dendrites (yellow arrowheads) and axons (white arrowheads). (E) Cross-sectioned retinal images showing fasciculation of eGFP-labeled axon from regenerated RGCs at optic disc at 14 days post NMDA injection. (F) Cross brain sections showing eGFP+ axon terminals of RGCs in the optic tectum at 14 days post light and NMDA damage. (G) Light-sheet images of whole-cleared brains showing regenerated RGCs projections into the optic tectum at 14 days post NMDA damage. Scale bars: A 100 um; B 200um; C 50 um, D 30 um; E 200um. LD: light lesion; dpl: days post lesion; NMDA: N-methyl-D-aspartate; dpi: days post injection; D: dorsal; V: ventral; N: nasal; T: temporal.GCL ganglion cell layer; INL: inner retinal layer; OPL: outer plexiform layer: ONL: outer nuclear layer.

We next examined the laminar positioning and connectivity of regenerated RGCs. In unlesioned retinas, RGC somata were confined to the GCL (Fig. 6A). In contrast, a substantial fraction of regenerated RGCs were ectopically positioned within the IPL and OPL in injured retinas (Fig. 6B, C). Quantification showed that 43.6% and 32.7% of eGFP+ regenerated RGCs are mislocalized in light-lesioned and NMDA-injected retinas, respectively. In light-lesioned retinas, 55% of these mislocalized regenerated RGCs are located in the OPL (Fig. 6B, C). These findings indicate that while RGCs identity is successfully specified during regeneration, precise laminar positioning is not fully restored.

We next asked whether regenerated RGCs re-establish long-range connectivity. In teleost fish, RGC axons project to the superficial layers of the optic tectum, the primary visual processing center. We observed eGFP+ RGCs extended their axons toward the optic disc and formed fasciculated bundles (Fig. 6D, E). Examination of the optic tectum revealed robust eGFP+ axonal projections within the stratum opticum at 14 dpi in both light-lesioned and NMDA-injected animals, whereas no eGFP signal was detected in unlesioned controls (Fig. 6F). In addition, tissue clearing combined with light-sheet 3D imaging showed that regenerated GFP RGC axons projected broadly across the optic tectum (Fig. 6G). Collectively, these data demonstrate that regenerated RGCs retain intrinsic axon guidance capacity and successfully re-establish distal projections, despite incomplete laminar precision within the retina.

## Discussion

A major challenge in regenerative neuroscience is not only to generate new neurons, but to rebuild the appropriate neuronal populations capable of restoring cellular diversity and circuit architecture. In the retina, recent studies have shown that Müller glia can be reprogrammed into neurons in mammals (Jorstad et al. 2017; Todd et al. 2022; Wohlschlegel et al. 2023; Le et al. 2024; Yang et al. 2025; Le et al. 2025; Hoang et al. 2020), yet the diversity, maturity, and circuit integration of regenerated neurons remain limited. Here, using lineage tracing, single-cell transcriptomics, and morphological analyses, we show that Müller glia–mediated regeneration in the adult zebrafish retina restores diverse neuronal types and key features of retinal circuit organization. Our results support four main conclusions. First, zebrafish Müller glia possess a robust regenerative capacity and can produce all major retinal cell types across injury paradigms. Second, injury context shifts the relative proportions of regenerated neurons without restricting overall neuronal diversity. Third, regenerated neurons largely converge on endogenous transcriptional identities, with minor differences that likely reflect ongoing maturation. Fourth, at the subtype levels, amacrine regeneration restores broad neurochemical and morphological diversity, while regenerated RGCs re-establish long-range projections to the optic tectum, demonstrating that adult zebrafish retain guidance cues sufficient for rebuilding circuitry. Together, these findings suggest that understanding how zebrafish achieve diverse lineage specification and integration may provide critical insight for reactivating regenerative potential in the human retina.

Our data show that Müller glia-derived progenitors generate all major retinal types while showing biased regeneration depending on the injury paradigm. The two lesion models used here selectively ablate different neuronal populations and produce corresponding shifts in the proportions of regenerated neurons. Although the mechanisms governing cell fate specification during regenerative neurogenesis remain to be elucidated, this process is likely regulated by the coordination of intrinsic developmental programs and extrinsic injury-induced signals. An intrinsic neurogenic program enables Müller glia-derived progenitors to generate multiple retinal lineages, whereas extrinsic injury-induced signals may bias cell lineages or cell survival toward the neuronal populations most affected by damage. This model explains why NMDA injury preferentially increases regeneration of inner retinal neurons, while light lesion promotes higher photoreceptor regeneration, yet neither paradigm restricts Müller glia-derived progenitors to a single fate. Previous transcriptomic studies have identified a shared core regenerative program activated in Müller glia across injury models, along with injury-specific responses (Hoang et al. 2020); (Lyu et al. 2023; Emmerich et al. 2023, 2024). Regardless of the initial transcriptomic changes, reprogrammed Müller glia produce multipotent Müller glia-derived progenitors and differentiate into various retinal neurons. Our data demonstrate the emergence of RGC markers prior to photoreceptor markers within eGFP-labeled neurons. Similarly, previous studies have shown that Müller glia-derived progenitors maintain conserved temporal expression patterns of developmental fate competence factors in Müller glia-derived progenitors following different lesion paradigms (Lahne, Brecker, et al. 2020). Together, these data suggest that regenerative neurogenesis largely recapitulates intrinsic developmental fate specification programs while remaining responsive to injury-specific signals. How the precise scale and proportion of regenerated neurons are regulated remains unclear, but inflammation appears to be an important modulator. We and others have previously demonstrated that elevated or prolonged inflammation promotes proliferation among Müller glia-derived progenitors and influences their fate specification (Silva et al. 2020; Nagashima and Hitchcock 2021; Magner et al. 2022). Whether different lesion types elicit a unique set of inflammatory signatures that can interact with intrinsic programs to direct specific lineages remains an important question for future studies.

Our data, together with prior work (Powell et al. 2016; Yoshimatsu et al. 2016), support the idea that Müller glia-derived progenitors are intrinsically multipotent and can produce “excess” neurons following both types of retinal damage. Our light lesion paradigm did not result in acute or robust death of inner retinal neurons, although we cannot fully exclude the possibility of minor secondary damage to the inner neurons. Recent work using nitroreductase-mediated selective ablation of RGC in larval retina, combined with pulse labeling of EdU, has reported prominent EdU staining in the GCL, but only rare EdU signals in other retinal layers, suggesting cell-specific regeneration (Emmerich et al. 2024). Although this study may suggest a difference in fate specification capacity between larval and adult retinas, the discrepancy may simply be due to the timing and dosage of EdU injection. The production of excess neurons has also been reported in multiple regenerative contexts, including spinal cord (Reimer et al. 2008), and may reflect an evolutionary strategy favoring robust neuronal replacement, with subsequent refinement through survival, maturation, and circuit integration. The fact that injury context shifts the proportions of regenerated neurons indicates that adjustment mechanisms exist, but they may be insufficient to precisely match the number and types of regenerated neurons to those that were lost. Elucidating how the system scales regenerative outcome, through progenitor proliferation, cell-fate specification, selective survival, or activity-dependent pruning, remains a key open question with direct implication in developing controllable regenerative strategy.

A common concern in regenerative biology is that newly generated neurons may express only a few lineage markers without acquiring fully mature programs. In our data, however, regenerated neurons show substantial transcriptional overlap with their endogenous counterparts across major retinal cell types. The modest differences observed are consistent with ongoing maturation, structural remodeling and circuit integration. For example, regenerated rods exhibit a relatively immature transcriptional profile at 14 dpi, including lower expression of mature phototransduction genes and persistence of immature markers. Likewise, regenerated RGCs maintain core identity transcription factors while upregulating genes associated with axon growth and guidance, consistent with neurons actively extending projections to re-establish connectivity. Together, these findings indicate that zebrafish regeneration produces bona fide neuronal identities that continue to mature over time.

RGC regeneration represents a stringent benchmark for circuit reconstruction, as it requires not only local synapse formation in the retina but also long-range axon projection to brain targets. We find robust eGFP+ axonal projections in the optic tectum after injury, demonstrating intrinsic axonal guidance mechanisms remain active in the adult zebrafish and are sufficient to support distal target connectivity. However, a subset of regenerated RGCs exhibited ectopic soma positioning in the IPL or OPL, suggesting incomplete laminar restoration and warranting future synaptic and physiological analyses to determine their circuit integration. Beyond RGCs, effective visual repair also depends on restoration of subtype diversity and laminar organization. By focusing on amacrine cells, one of the most diverse retinal cell types, we show that multiple subtypes across major neurochemical classes are regenerated in both injury paradigms. These regenerated cells not only re-establish appropriate gene expression profiles but also recover their characteristic morphology. For example, Chat+ starburst amacrine cells reform their typical radial dendritic arbors, and TH+ dopaminergic amacrine cells extend processes that integrate into existing retinal circuits. Together, these results indicate that the adult zebrafish retina retains instructive cues necessary to guide proper subtype specification, dendritic patterning and circuit integration. These observations have important translational implications. Although spontaneous regeneration in mammals is limited, recent studies from our group (Yang et al. 2025) and others (Jorstad et al. 2017; Todd et al. 2022) have shown that neurons generated from Müller glia can respond to light stimulation, indicating that elements of circuit plasticity persist in the adult mammalian retina. Our results position the zebrafish retina for high-fidelity neuronal regeneration and highlight the need to identify and activate the molecular programs to unlock regeneration in humans.

During retinal development, cone genesis occurs around 72 hours post fertilization, whereas rod genesis continues throughout life from fate-restricted rod progenitors located in the ONL of the mature retina (Raymond and Rivlin 1987; Bringmann et al. 2006). Although these rod precursors originate from Müller glia(Bernardos et al. 2007), our mmp9:creERT2 system does not label rod precursor-derived cells in the absence of injury. Rod precursors are known to replenish rods following minor or chronic photoreceptor damage, but only when extent of damage remains below the threshold required to trigger Müller glia reprogramming (Morris et al. 2008; Montgomery et al. 2010; Fogerty et al. 2022; Shihabeddin et al. 2025). Our results therefore indicate that the regenerated rod photoreceptors observed after light lesion or NMDA injury primarily arise from reprogrammed Müller glia rather than from pre-existing rod precursors. These regenerated rods likely originate either directly from the initial asymmetric division of Müller glia or from Müller glia–derived progenitors that subsequently generate rod precursors.

The injury-inducible lineage tracing line used in this study was originally developed to lineage trace regenerated osteoblast (Ando et al. 2017). The mmp9 promoter, which is rapidly and exclusively induced in reprogrammed Müller glia following retinal injury (Silva et al. 2020), enables specific labeling of the subset of Müller glia that enter the regenerative program, in contrast to pan-glial promoters such as *gfap* that label all Müller glia. This approach allows for the identification and analysis of regenerated neurons derived from Müller glia. One limitation of this transgenic system is that the intensity of the actin-driven eGFP reporter varies across cell types, resulting in strong labeling in RGCs but weaker signals in photoreceptors and some interneurons. Future studies using optimized reporters or cell type–specific labeling strategies may further improve morphological and functional characterization of regenerated neurons.

This study has several limitations. First, our analyses focus primarily on molecular identity and morphology at a relatively early regeneration stage (14 dpi), and therefore may not fully capture the long-term maturation and functional integration of regenerated neurons. Future studies using electrophysiological and behavioral assays will be necessary to determine whether these neurons restore visual function. Second, the number of regenerated cells recovered for scRNA-Seq following light and NMDA injuries was relatively limited, which may reduce the power to detect subtle differences in cell-type composition or transcriptional states. Third, although we performed detailed subtype analysis of amacrine cells, similar high-resolution analyses of other retinal populations, including bipolar cells, photoreceptors, and RGC subtypes, could be performed to fully evaluate the diversity of regenerated neurons. Finally, our injury paradigms model acute neuronal loss and may not fully reflect the complexity of retinal degeneration observed in retinal diseases.

In summary, our findings demonstrate that Müller glia–mediated regeneration in the adult zebrafish retina restores neuronal diversity, subtype identity and key features of circuit architecture. Our study establishes zebrafish retinal regeneration as a valuable model for understanding how complex neuronal diversity and connectivity can be rebuilt after injury and provides important insights for developing regenerative strategies in the mammalian retina.

## Methods

### Zebrafish.

Fish were maintained at 28C on a 14/10 h/ light/ dark cycle with standard husbandry procedures. Adults were of either sex and used between 4–10 months of age. To generate lineage tracing lines, male Tg(mmp9:creERt2)tyt208 (Ando et al. 2017) were out crossed with female Tg(Olactb:loxP-dsred2-loxP-egfp)tyt201Tg (Yoshinari et al. 2012). To avoid premature recombination in the germline or early embryonic stage due to maternal deposition of Cre mRNA/protein, male Cre-drivers were used. All experimental protocols were approved by the University of Michigan Institutional Animal Care and Use Committee.

### Pharmacological treatment.

To induce Cre-mediated recombination, zebrafish were treated with 4-hydroxytamoxifen (4-OHT) via immersion. A stock solution of 2.5mM 4-OHT were prepared in Ethanol and stored at −20C. For treatment, the stock was diluted in system water to a final concentration of 2.5uM. Adult fish were subjected to four consecutive, 12 hours of overnight treatments (Pinzon-Olejua et al. 2017). In between each treatment, animals were placed in a system of water. To prevent photodegradation of the Tamoxifen, tanks were kept in the dark during the incubation periods. To assay cell proliferation, EdU (5-Ethynyl-2’-deoxyuridine, Thermo Fisher Scientific) was administered intraperitoneally. Fish were anesthetized in 0.02% tricaine methane sulfonate. 20 uL of 1mg/ml EdU in PBS was injected intraperitoneally using a 30Gx1/2 needle with 1ml syringe at 24, 48, 72, and 96 hours post lesion.

### Retinal lesions.

To selectively damage photoreceptors, an intense light lesion was used (Bernardos et al. 2007; Taylor et al. 2012). Zebrafish were exposed to 100,000 lux light from an EXFO X-Cite 120 W metal halide lamp for 30 min. To damage inner retinal neurons, 0.5uL of 100 mM NMDA in PBS was injected intravitreally. Fish were anesthetized in 0.02% tricaine methane sulfonate. Using a 30Gx1/2 needle, a small incision was made in the cornea. A hamilton syringe equipped with a blunt 30G needle was carefully inserted behind the lens, then 0.5 ml of NMDA was delivered to the vitreous.

### Fixation and Tissue processing.

Following anesthesia in 0.02% tricaine methanesulfonate, eyes were enucleated and placed in 4% paraformaldehyde in 0.1M phosphate buffer (pH7.4) overnight at 4C. After rinsing with 5% sucrose solution, tissues were cryoprotected with 20% sucrose solution, then embedded in OCT/ 20% sucrose solution. Retinal sections (6mm) were cut on a cryostat.

### Immunohistochemistry.

Immunohistochemistry was performed as described in our previous studies (Nagashima et al. 2020; Silva et al. 2020). Briefly, tissues were fixed in 4% paraformaldehyde in 5% sucrose/0.1M Phosphate buffer (pH 7.4) overnight at 4C. Immunohistochemistry was performed on 6 mm retinal sections as described previously. Briefly, sections were blocked in blocking reagents containing 20% normal goat serum/0.5% Triton X-100 in pH7.4, with 0.1% sodium azide. Primary antibodies (Table 1) were diluted in a dilution buffer containing 1% normal goat serum/0.5% Triton X-100 in pH7.4, with 0.1% sodium azide and incubated 4C overnight. AlexaFluor secondary antibody (Thermo Fisher Scientific) incubation was performed at room temperature for 2 hours. Slides were stained with Hoechst 33342 (Thermo Fisher Scientific) for nuclear staining. Sections were mounted with ProLong Gold (Thermo Fisher Scientific). For goat anti-Choline acetyltransferase antibody, horse serum was utilized as the blocking and dilution agents in place of goat serum to prevent cross-reactivity. To perform flat-mount immunocytochemistry, retinas were isolated from the eyes of dark-adapted zebrafish and fixed overnight at 4C in 4% paraformaldehyde in 5% sucrose/ 0.1M Phosphate buffer (pH 7.4). Immunohistochemistry was performed as previously described (Silva et al. 2020; Nagashima et al. 2020) using blocking (10% normal goat serum/1% Tween 20/1% Triton X-100/1% DMSO in PBS, pH 7.4, with 0.1% sodium azide) and dilution (0.5% normal goat serum/1% Tween 20/1% Triton X-100/1% DMSO in PBS, pH 7.4, with 0.1% sodium azide) reagents.

### EdU and TUNEL assays.

Visualization of EdU labeled cells and TUNEL staining were performed using Click-iT EdU Alexa Fluor 647 Imaging kit (Thermo Fisher Scientific), and In Situ Cell Death Detection Kit, TMR red (Millipore Sigma), respectively, according to the manufacturer’s instructions. For TUNEL staining, slides were treated with PBS containing 1% sodium citrate/ 1% Triton X-100 at 4C. After rinsing with PBS, slides were incubated with a TUNEL reaction cocktail for 30 min at 37C. For EdU detection, freshly prepared Click-iT reaction cocktails were treated for 30 minutes at room temperature.

### Imaging and analysis.

Fluorescence images of retinal sections were captured using Leica Stellaris 8 Falcon Confocal Microscope. Flat-mount retinal preparations were imaged using Leica SP5 Confocal Microscope. Cell counts were performed on Leica Application Suite X (Leica Microsystems). For retinal cross-section, cell counts were quantified across a 300 mm linear length of the retina. To ensure representative sampling, 2–3 non-adjacent sections were analyzed per retina. The resulting counts were averaged per individual and normalized. For flat-mount preparation, cells in 22,500um2 were quantified and normalized.

### Retinal dissociation, FACS-sorting and scRNA-Seq.

Retinas were dissected in ice cold Hibernate A (Gibco A1247501) and dissociated using the Papain Dissociation System (LK003150, Worthington) with the following modifications. Retinas were incubated in papain enzyme buffer at 4C for 20min, then 28C for 30 min, with tube mixing by inversion every 5 min. The tissue was centrifuged at 200×g for 2 min and papain solution removed, then dissociated in ice cold Hibernate-A buffer by trituration. The single cell suspension was subjected to gradient centrifugation through ovomucoid albumin at 200×g for 7min to exclude dead cells. Live pelleted cells were resuspended in HBAG buffer containing Hibernate A, B 27 supplement (Thermo Fisher 17504044), and GlutaMAX (Thermo Fisher 35050061), filtered through 50um filter, and then subjected to FACS using a Sony MA900 cell sorter for GFP+ cells into HBAG buffer. Sorted cells were mixed with BSA at 0.3% final concentration to reduce potential cell clumping before centrifugation at 400×g for 5min to concentrate, then resuspended in a desired volume of HBAG buffer to reach a concentration of 500–1500 cells/uL. Cell viability and counts were determined via trypan blue staining and haemocytometer. Cells were submitted to the University of Michigan Advanced Genomics Core for 3’ scRNA-Seq. GFP+ cells (~15k) were loaded into the 10X Genomics Chromium Single Cell System and libraries were generated using V4 chemistry following the manufacturer’s instructions. Libraries were sequenced on the Illumina NovaSeq platform (500 million reads per library).

### scRNA-Seq analysis.

Single-cell RNA-sequencing data were analyzed using the Scanpy framework (v1.9.3) together with AnnData (v0.12.8). Quality control filtering was applied to remove low-quality cells and potential multiplets. Cells were retained if they expressed more than 800 and fewer than 6,000 genes, contained between 1,200 and 30,000 total transcripts, and exhibited less than 25% mitochondrial gene expression. In addition, cells expressing fewer than 100 genes were excluded, and genes detected in fewer than three cells were removed from further analysis. Following quality control, gene expression values were normalized to account for differences in sequencing depth and log-transformed. Highly variable genes were identified and used for downstream analyses. Dimensionality reduction was performed using principal component analysis to capture the major sources of transcriptional variation. To correct for batch effects between samples, data integration was performed using the Harmony algorithm. Batch-corrected principal components were used to construct a nearest-neighbor graph, which served as the basis for downstream analyses. Cell populations were identified using graph-based clustering with the Leiden algorithm.

Following initial clustering and cell type annotation, specific cell populations were subset based on annotated cell type identity. Cells belonging to selected cell types were extracted from the integrated dataset and re-analyzed independently to enable higher-resolution characterization of within-population heterogeneity. Differential gene expression analysis was performed using a nonparametric Wilcoxon rank-sum test as implemented in Scanpy. Depending on the analysis context, differential expression was assessed either between annotated cell clusters or between specified sample groups. For cluster-level analyses, genes were ranked for each cluster relative to all remaining cells, whereas sample-level comparisons were performed between cells derived from two selected samples. For each comparison, gene-level statistics including Wilcoxon scores, log fold changes, and multiple-testing–adjusted p-values were calculated. Differentially expressed genes were ranked based on statistical significance or effect size and used for downstream visualization and interpretation.

## Supplementary Material

Supplement 1 Supplementary Figure 1. Cell type-specific ablation of retinal neurons following light lesion or NMDA injection.
(A) TUNEL staining on retinal sections at 24 hours post light lesion. (B) Quantification of TUNEL+ cells in different retinal layers at 24, 48, and 72 hours post light lesion. (C, D) Immunocytochemistry and quantification of HuC/D+ neurons in the ganglion cell layer and the inner nuclear layer at 0, 48, and 72 hours post light lesion. (E) TUNEL staining at 24 hours post NMDA injection. (F) Quantification of TUNEL+ cells in different retinal layers at 24, 72hr, and 5 days post NMDA injection.ONL: outer nuclear layer; INL: inner nuclear layer; GCL ganglion cell layer. Scale bar = 50um.
Supplementary Figure 2. Validation for specificity and temporal analysis of Tg(mmp9:creERT2; Ola.actb:lox-p-dsRed-loxp-eGFP) lineage tracing line after injuries.
(A) Experimental paradigm and retinal sections immunostained for eGFP following 4-hydroxitamoxifen (4-OHT) treatment without retinal injury. (B) Experimental paradigm and retinal sections immunostained for eGFP following retinal injury without 4-OHT treatment. (C) Immunocytochemistry for Müller glial marker, glutamine synthetase (GS), at 3 days post light lesion. (D, E) Immunocytochemistry for cone (Zpr1, D) and rod (Zpr3, E) photoreceptor markers at 7 days post light lesion. Scale bars 50 um; ONL: outer nuclear layer; INL: inner nuclear layer: GCL: ganglion cell layer; LD: light lesion; dpl: days post lesion; dpi: days post injection; D: dorsal; V: ventral; N: nasal; T: temporal.
Supplementary Figure 3. Immunohistochemical validation of regenerated retinal neurons following NMDA damage.
Representative immunostained images for eGFP and rod (Zpr3), cone (Zpr1), bipolar cells (Cabp5), amacrine cells (HuC/D), horizontal cells and retinal ganglion cells (Rbpms) at 14 days post NMDA injected retinas. NMDA: N-methyl-D-aspartate; dpi: days post injection;onl:outer nuclear layer; inl: inner nuclear layer: gcl: ganglion cell layer. Scale bar 50 um.
Supplementary Figure 4. ScRNA-Seq analysis and differentially expressed gene analysis of control and regenerated neurons
Volcano plots representing differentially expressed genes, and boxed plots of selected genes in rod (A), amacrine cells (B, C), bipolar cells (D,E), and horizontal cell (F,G) and RGC (H) populations. LD: light lesion; NMDA: N-methyl-D-aspartate; BC: bipolar cells; AC: amacrine cells; HC: horizontal cells.
Supplementary Figure 5. ScRNA-Seq analysis of amacrine clusters across different sample groups
(A). UMAP plots showing amacrine cell clusters separated by sample groups. (B) Dot plot showing top 2 expressed genes across 45 distinct amacrine clusters. (C) Proportion of 45 amacrine subtypes in unlesioned, light lesioned and NMDA injected samples.
Supplementary Figure 6. Regenerated amacrine cells show diverse neurochemical signatures.
(A) UMAP feature plot showing expression of glycinergic amacrine cell marker, slc6a9. (B) Proportion of subtypes across different samples. (C) Immunocytochemistry for amacrine cell subtype markers, Gad65/67, TH, and Chat (magenta) at day 14 after NMDA damage. Scale bars: 50 um. LD: light lesion; ONL:outer nuclear layer; INL: inner nuclear layer; GCL: ganglion cell layer.

Supplement 2 **Table 1.** Key reagents used in the study

## Data Availability

Raw scRNA-Seq data is available at Gene Expression Omnibus under accession number GSE319801. Codes used for this study is available at our GitHub repository: https://github.com/SherineAwad/Zebrafish_scRNASeq_scanpy
