# Supplementary material for: Müller glia–mediated regeneration restores neuronal diversity and retinal circuit organization in the adult zebrafish
Source: bioRxiv. 2026 Mar 17:2026.03.15.711785. Preprint. [Version 1] doi: 10.64898/2026.03.15.711785 (PMC13015437; doi:10.64898/2026.03.15.711785)
Supplement: Supplement 1 — Supplementary Figure 1. Cell type-specific ablation of retinal neurons following light lesion or NMDA injection. (A) TUNEL staining on retinal sections at 24 hours post light lesion. (B) Quantification of TUNEL+ cells in different retinal layers at 24, 48, and 72 hours post light lesion. (C, D) Immunocytochemistry and quantification of HuC/D+ neurons in the ganglion cell layer and the inner nuclear layer at 0, 48, and 72 hours post light lesion. (E) TUNEL staining at 24 hours post NMDA injection. (F) Quantification of TUNEL+ cells in different retinal layers at 24, 72hr, and 5 days post NMDA injection.ONL: outer nuclear layer; INL: inner nuclear layer; GCL ganglion cell layer. Scale bar = 50um. Supplementary Figure 2. Validation for specificity and temporal analysis of Tg(mmp9:creERT2; Ola.actb:lox-p-dsRed-loxp-eGFP) lineage tracing line after injuries. (A) Experimental paradigm and retinal sections immunostained for eGFP following 4-hydroxitamoxifen (4-OHT) treatment without retinal injury. (B) Experimental paradigm and retinal sections immunostained for eGFP following retinal injury without 4-OHT treatment. (C) Immunocytochemistry for Müller glial marker, glutamine synthetase (GS), at 3 days post light lesion. (D, E) Immunocytochemistry for cone (Zpr1, D) and rod (Zpr3, E) photoreceptor markers at 7 days post light lesion. Scale bars 50 um; ONL: outer nuclear layer; INL: inner nuclear layer: GCL: ganglion cell layer; LD: light lesion; dpl: days post lesion; dpi: days post injection; D: dorsal; V: ventral; N: nasal; T: temporal. Supplementary Figure 3. Immunohistochemical validation of regenerated retinal neurons following NMDA damage. Representative immunostained images for eGFP and rod (Zpr3), cone (Zpr1), bipolar cells (Cabp5), amacrine cells (HuC/D), horizontal cells and retinal ganglion cells (Rbpms) at 14 days post NMDA injected retinas. NMDA: N-methyl-D-aspartate; dpi: days post injection;onl:outer nuclear layer; inl: inner nuclear layer: gcl: ganglion cell [file media-1.pdf]

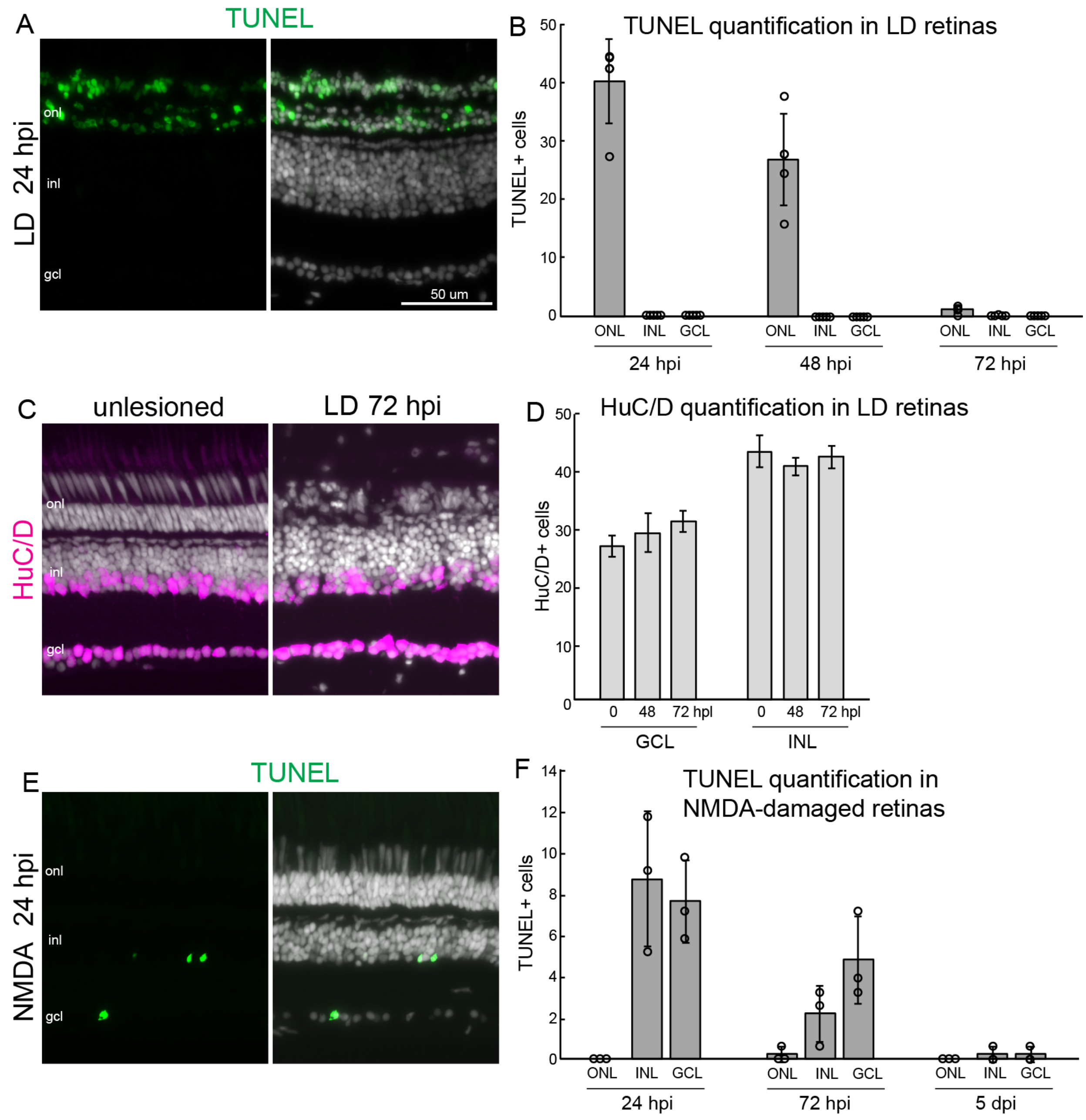

Supplementary Figure 1. Cell type-specific ablation of retinal neurons following light lesion or NMDA injection.(A) TUNEL staining on retinal sections at 24 hours post light lesion. (B) Quantification of TUNEL+ cells in different retinal layers at 24, 48, and 72 hours post light lesion. (C, D) Immunocytochemistry and quantification of HuC/D+ neurons in the ganglion cell layer and the inner nuclear layer at 0, 48, and 72 hours post light lesion. (E) TUNEL staining at 24 hours post NMDA injection. (F) Quantification of TUNEL+ cells in different retinal layers at 24, 72hr, and 5 days post NMDA injection.ONL: outer nuclear layer; INL: inner nuclear layer; GCL ganglion cell layer. Scale bar = 50um.

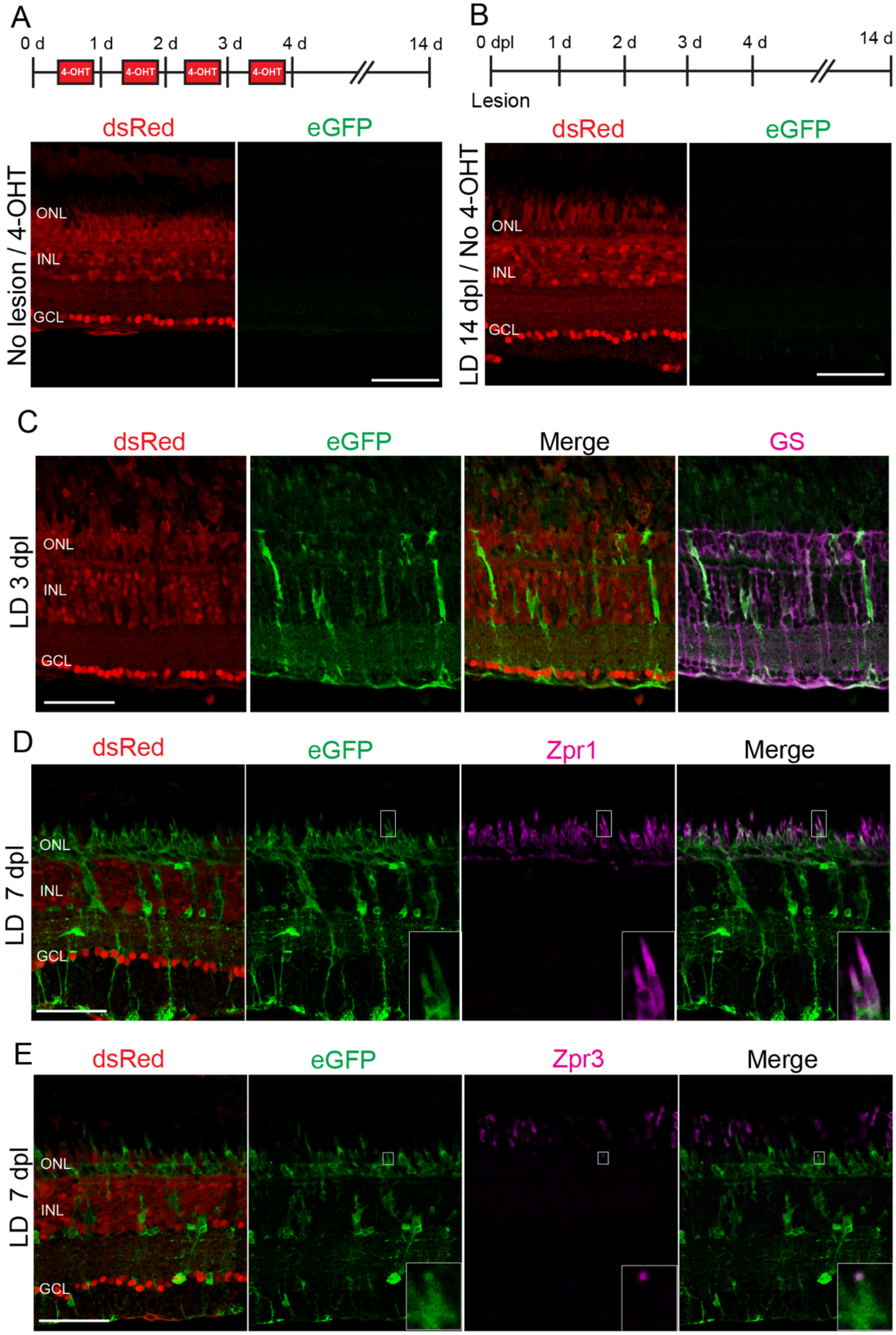

Supplementary Figure 2. Validation for specificity and temporal analysis of Tg(mmp9:creERT2; Ola.actb:lox-p-dsRed-loxp-eGFP) lineage tracing line after injuries. (A) Experimental paradigm and retinal sections immunostained for eGFP following 4-hydroxitamoxifen (4-OHT) treatment without retinal injury. (B) Experimental paradigm and retinal sections immunostained for eGFP following retinal injury without 4-OHT treatment. (C) Immunocytochemistry for Müller glial marker, glutamine synthetase (GS), at 3 days post light lesion. (D, E) Immunocytochemistry for cone (Zpr1, D) and rod (Zpr3, E) photoreceptor markers at 7 days post light lesion. Scale bars 50 um; ONL: outer nuclear layer; INL: inner nuclear layer: GCL: ganglion cell layer; LD: light lesion; dpl: days post lesion; dpi: days post injection; D: dorsal; V: ventral; N: nasal; T: temporal.

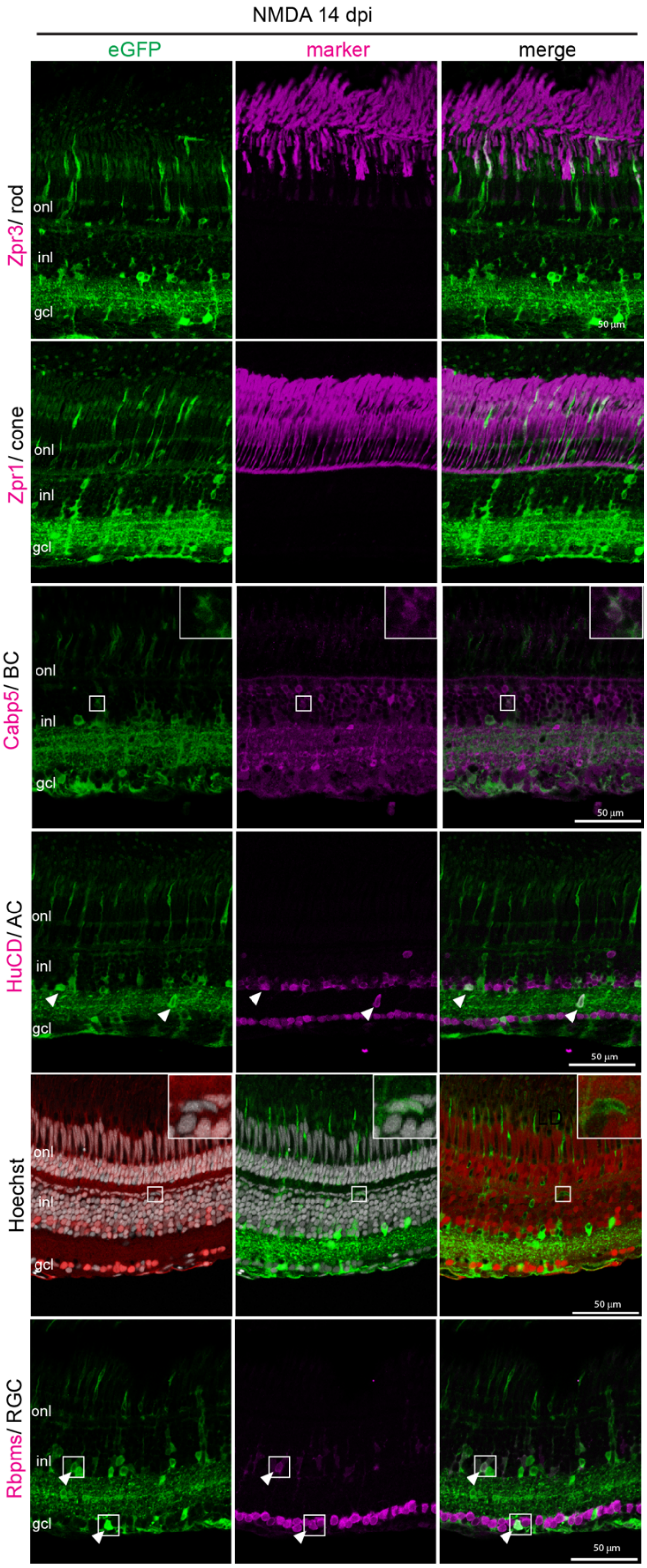

Supplementary Figure 3. Immunohistochemical validation of regenerated retinal neurons following NMDA damage. Representative immunostained images for eGFP and rod (Zpr3), cone (Zpr1), bipolar cells (Cabp5), amacrine cells (HuC/D), horizontal cells and retinal ganglion cells (Rbpms) at 14 days post NMDA injected retinas. NMDA: N-methyl-D-aspartate; dpi: days post injection;onl:outer nuclear layer; inl: inner nuclear layer: gcl: ganglion cell layer. Scale bar 50 mm.

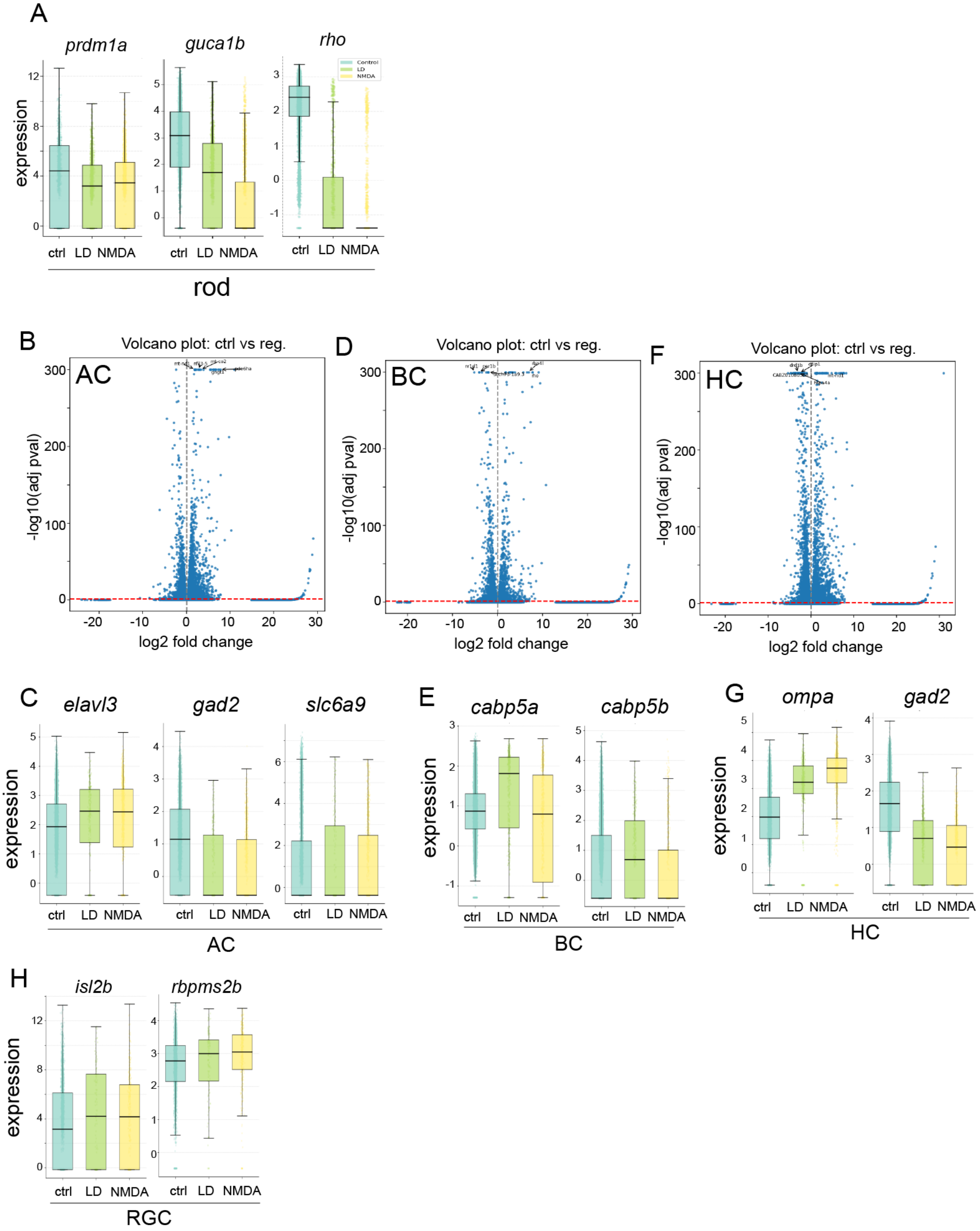

Supplementary Figure 4. ScRNA-seq analysis and differentially expressed gene analysis of control and regenerated neurons. Volcano plots representing differentially expressed genes, and boxed plots of selected genes in rod (A), amacrine cells (B, C), bipolar cells (D,E), and horizontal cell (F,G) and RGC (H) populations. LD: light lesion; NMDA: N-methyl-D-aspartate; BC: bipolar cells; AC: amacrine cells; HC: horizontal cells.

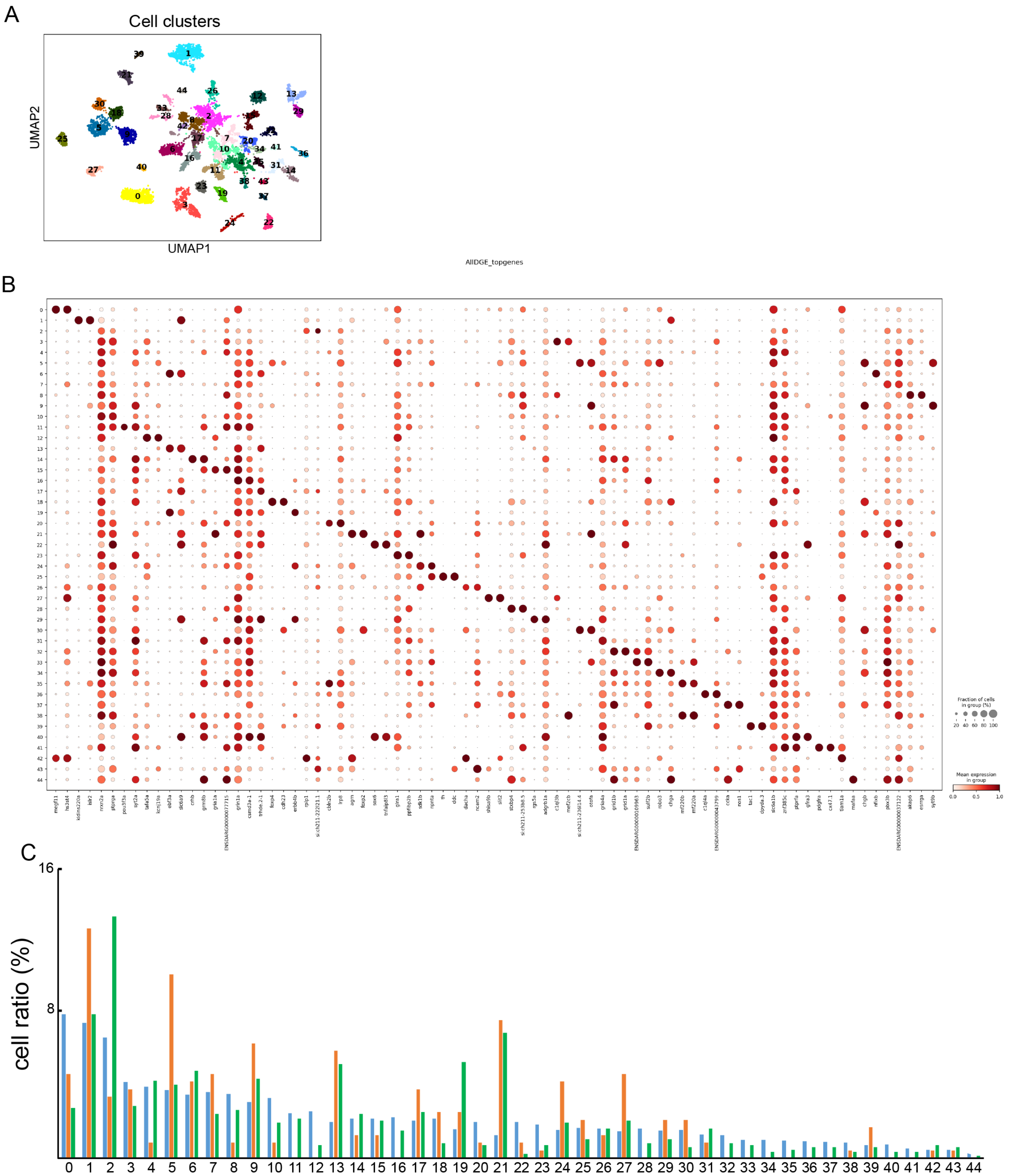

Supplementary Figure 5. ScRNA-Seq analysis of amacrine clusters across different sample groups (A). UMAP plots showing amacrine cell clusters separated by sample groups. (B) Dot plot showing top 2 expressed genes across 45 distinct amacrine clusters. (C) Proportion of 45 amacrine subtypes in unlesioned, light lesioned and NMDA injected samples.

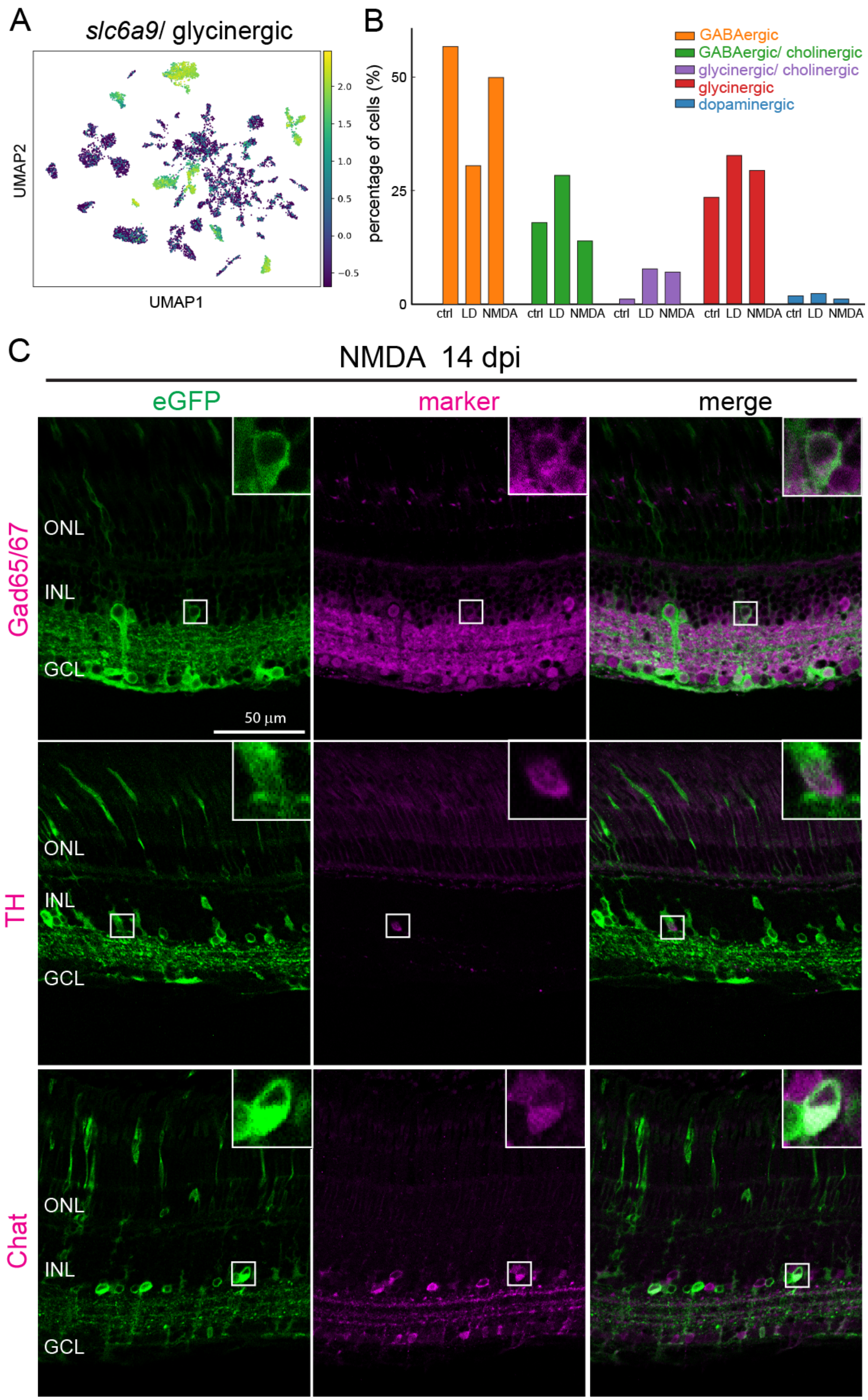

Supplementary Figure 6. Regenerated amacrine cells show diverse neurochemical signatures after NMDA injury. (A) UMAP feature plot showing expression of glycinergic amacrine cell marker, slc6a9. (B) Proportion of subtypes across different samples. (C) Immunocytochemistry for amacrine cell subtype markers, Gad65/67, TH, and Chat (magenta) at day 14 after NMDA damage. Scale bars: 50 mm. LD: light lesion; ONL:outer nuclear layer; INL: inner nuclear layer; GCL: ganglion cell layer.
